# Tracing innovation pathways behind fisheries co-management in Vanuatu

**DOI:** 10.1007/s13280-022-01788-y

**Published:** 2022-09-22

**Authors:** Dirk J. Steenbergen, Jacob Raubani, Sompert Gereva, William Naviti, Christopher Arthur, Ajay Arudere, Jayven Ham, Lucy Joy, Watisoni Lalavanua, Pita Neihapi, Akiya Seko, Hiroaki Terashima, Neil L. Andrew

**Affiliations:** 1 Australian Centre for Ocean Resources and Security (ANCORS), University of Wollongong (UOW), North Wollongong, Wollongong, NSW 2500 Australia; 2 Pacific Islands Forum Fisheries Agency (FFA), Honiara, Solomon Islands; 3 Vanuatu Fisheries Department, Mingkai Building, Teoma Street, PO Box 9045, Port-Vila, Vanuatu; 4 Pacific Community-Fisheries Aquaculture and Marine Ecosystem Division (SPC-FAME), CPS B.P. D5, 98848 Nouméa, New Caledonia; 5 IC Net Limited, Land Axis Tower, 27th Floor 11-2 Shintoshin, Chuo-ku, Saitama-shi, Saitama 330-6027 Japan

**Keywords:** Community-based fisheries management, Governance, Innovation, Small-scale fisheries, Theory of scaling, Vanuatu

## Abstract

Co-management approaches have become a core part of coastal fisheries policy and planning practice in Vanuatu. With a long history of supporting community based fisheries management (CBFM), we trace its evolution in Vanuatu to understand how new structures and processes become adopted at scale. A theory of scaling for CBFM guides the analysis of regime shifts over time. We discuss planning for sustained spread under a national programme by categorising multiple drivers of change through three intervention pathways focussed, respectively, on developing (i) an enabling environment, (ii) institutional and individual capacity, and (iii) focussed innovative action in smaller targeted constituencies. Whilst we argue that local fisheries co-management institutions balance competing interests, and so differ amongst places, we also recognise the importance of connectivity and continuity. The realisation of a national programme therefore requires patchworks of siloed projects to be knitted together into coordinated programmatic approaches that strategically integrate activities.

## Introduction

Small-scale fisheries drive far-reaching fish trade and distribution networks, provide food security and income, are cultural cornerstones in local custom and identity, and strengthen resilience for the thousands of remote coastal communities of the Pacific region and beyond (Cohen et al. 2019). These fisheries require effective management to maintain productivity in the face of many external (socio-political, economic, and/or physical) drivers of change. To overcome the otherwise insurmountable challenge of managing vast expanses of coastlines through central state administrations, under-resourced governments are deploying co-management approaches to harness local capacities and knowledge that can more effectively manage coastal resources across extensive, remote coastal seascapes (e.g. Schwarz et al. 2021).

The essence of these decentralised approaches involves sharing of management authority and responsibility between communities and government and/or non-government agents. This division may vary on a spectrum from community-driven to externally driven arrangements (Carlsson and Berkes 2005). Research in the last decade has helped clarify the conditions that are conducive to effective collective action, and thus also co-management (Agrawal 2002). Much of this work has drawn on small-scale, context-specific cases, but the development challenges facing countries in the Pacific and elsewhere today require effective management to be widespread and interconnected, rather than isolated across a few select communities.

One of the region’s foremost policy recommendations on community-based fisheries management (CBFM), ‘A New Song for Coastal Fisheries in the Pacific’, states explicitly that “[s]mall pockets of effective coastal fisheries management will not be adequate to address the problem. Ways must be found of building on successes and expanding them to meaningful proportions of the coastal environment” (Pacific Community 2015, p. 5). Pacific Island Countries and Territories (PICTs) are therefore challenged to scale collective action institutions like CBFM to ensure that coastal fisheries can continually and reliably provide food and support household incomes in the face of various changes. In response to this, PICTs endorsed the Pacific Framework for Action on Scaling up CBFM: 2021–2025 (hereafter the Regional Scaling Framework, Pacific Community 2021). The Regional Scaling Framework provides principles and guidance to assist national fisheries agencies, and their non-government partners and stakeholders to scale CBFM across a great number of coastal communities.

These regional initiatives exist within a diverse array of national fisheries governance and regulatory arrangements. Whilst some countries have long histories of engagement with co-management, for others such collaborative arrangements are in their infancy. Regardless of the maturity of national programmes, it is important to consider that *scaling* is an integral part of CBFM development within a country, or specific jurisdiction, and not as a subsequent stage of development once CBFM has been innovated, refined, and deemed *ready* to bring to scale (Wigboldus and Leeuwis 2013; Sartas et al. 2020). This insight has implications for how we understand development trajectories, and where scaling happens as part of those trajectories, i.e. understanding that CBFM scaling processes evolve through concerted and intentional actions, as well as unintended and unforeseen changes or disruptions.

Whereas ‘scaling’ has been assessed in land-based agrarian studies where technology-based innovations often feature centrally in analysis (Wigboldus and Leeuwis 2013), few scholars have applied a theory of scaling to collective action institutions in the coastal fisheries sector. Tracing uptake of technological innovations is after all very different from tracing how collective action institutions become widespread. We build on Steenbergen et al.’s (2022) theoretical framing to analyse processes through which CBFM policies and practices have evolved in Vanuatu. In doing so, we contribute to broadening the literature on scaling agricultural and natural resource management innovations.

The paper seeks to better understand the current shape and function of Vanuatu's regime of coastal fisheries management. Many small ‘experimental’ initiatives have consolidated over time to influence policy and practice towards mainstream CBFM application. We use scaling theory for CBFM to frame developments in coastal fisheries management, allowing for identification of opportunities and approaches that may sustain local collective action across constituencies. In the following sections, we introduce Vanuatu’s coastal fisheries context, before outlining the conceptual underpinnings of a theory of scaling for CBFM used to frame our discussion. As part of our results, we present the growth of CBFM and evolution of a national programme in Vanuatu, using published material tracing back over 30 years of policies and local experience. We draw out implications for institutionalising CBFM in Vanuatu and examine potential coordination and implementation strategies involving government, non-government, and civil society stakeholders. In applying the framework, insights about how institutionalisation may occur are presented through three main intervention pathways in our discussion; ‘enabling environment’, ‘communication and information’, and ‘co-management’.

## Methods

The paper builds on a critical review of peer-reviewed, published, and unpublished material, to chart the growth of CBFM in Vanuatu (see Muilerman et al. 2018 for an example of the applied methodology). Applying Steenbergen et al. (2021)’s framework, innovation trajectories are mapped over ‘levels of structuration’ and ‘phases of scaling’ that cumulatively result in governance regime re-configurations. In populating the framework we draw from, and update, published reviews, for example, on relevant policy in the region by Govan (2018) and on CBFM programmes and projects in Vanuatu by Raubani et al. (2017). In addition, we qualitatively analyse documentation of CBFM activities through reports, media articles, and workshop outputs to inform the chronological regime change. The authors’ experience and direct involvement in the policy and practice processes at different phases of CBFM development, both regionally and in Vanuatu, guided the qualitative assessment.

### Coastal fisheries and their management in Vanuatu

**Fig. 1:**
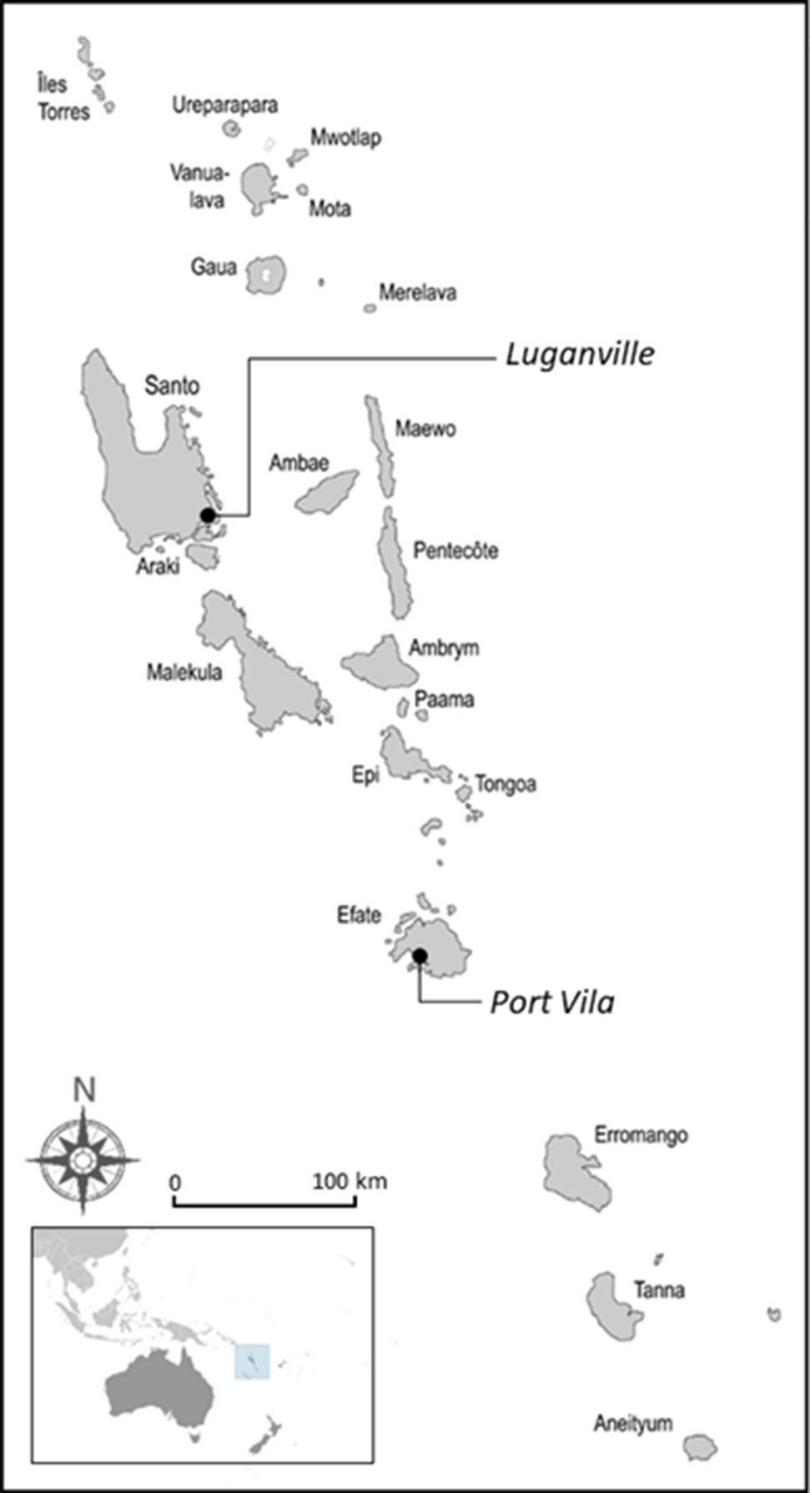
Map of Vanuatu indicating major islands and the two urban centres of Port Vila (national capital) and Luganville

Vanuatu has an exclusive economic zone (EEZ) of ca. 690 000 km^2^ with a coastline of 3000 km that adjoins a total estimated reef area of 1200 km^2^; primarily fringing reefs. Just 2% of the EEZ is made up by the more than 80 small islands strung in a north–south orientation (Fig. 1). Unsurprisingly, coastal fisheries are an important part of everyday life for the population of ca. 300 000 ni-Vanuatu (SPC 2022). Located in the southern sub-tropical cyclone-belt and along the geothermally active tectonic fault line known as the Pacific ‘Ring of Fire’, disruptions resulting from extreme weather events, volcanos and earthquakes are frequent and intense (Vanuatu's society ranks as the world's most vulnerable to natural disasters, Aleksandrova et al. 2021). In this context coastal fisheries’ contributions to ensuring rural food security have been demonstrated widely, particularly in times of hardship (e.g. where natural disasters have higher impact on terrestrial food supply than marine ones) and/or high demand (e.g. spikes in communal demands for food related to social events like funerals) (Pakoa et al. 2019; Steenbergen et al. 2020).

Whilst offshore tuna fisheries provide significant national income, coastal small-scale fisheries drive thriving domestic trade and consumption. Inshore fisheries provide a critical source of protein and micronutrients in the otherwise poor diet of many ni-Vanuatu (Vanuatu National Statistics Office 2007). High value species, including deep water snappers, groupers, and invertebrates (e.g. lobster, freshwater prawns), are targeted specifically for high-end consumption, including the international tourism market.

Throughout Vanuatu, customary (*kastom*) structures and systems, are strongly adhered to and manifest themselves through tenure arrangements, fishing restrictions around sacred sites, and cultural beliefs and practices shaping people’s interactions with their resources and environments (Vierros et al. 2010). Such arrangements are enshrined in the national constitution (Art 73: Chap 12), which acknowledges customary tenure, extending to outer reefs. Consequently, there is a myriad of civil society and/or community-based stakeholders who play important roles, most significant of which are local leaders and their communities. In this context the Vanuatu Fisheries Department (VFD), holds the overarching mandate to support management, operationalise development targets as defined in the National Sustainable Development Plan (‘The People’s Plan’) and enforce regulations over maritime zones in Vanuatu (including inshore waters and coastal zones). VFD utilises nationally allocated budgets in combination with funds secured through bilateral development programmes to ensure investments are multi-scale, implementation of management is decentralised, and grass-roots governance agents are empowered.

**Fig. 2:**
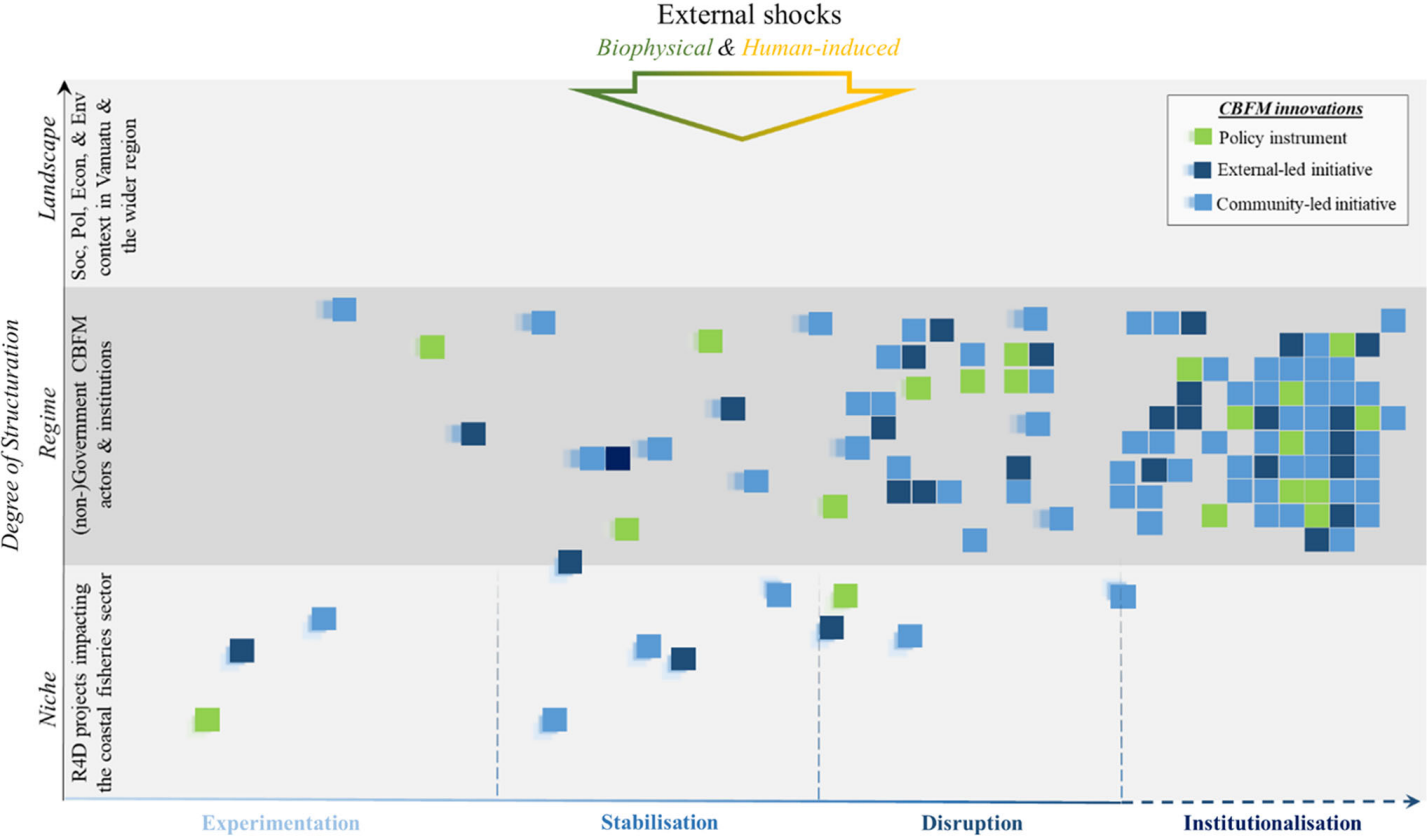
CBFM scaling framework providing a heuristic tool to map multiple CBFM innovation trajectories across a policy and practice arena, adapted from Steenbergen et al. (2022)

Local collective action that promotes or enables sustainable fishing can be self-initiated or enabled with external support; with the former involving management that communities are doing independently (e.g. customary closures over marine areas) and the latter ranging from information provision activities to interventions in communities (e.g. co-developing management plans). Another category of CBFM initiative involves actions that enable CBFM, such as policy and legislation. The thrust of CBFM initiatives can therefore be seen to empower communities, build capacity in communities, and/or integrate local actors into larger (national) governance structures. Table 1 outlines some key attributes associated with CBFM initiatives (such initiatives feature as different coloured squares on trajectories that shape a regime change in Figs. 2, 4, 5).

**Table 1:**
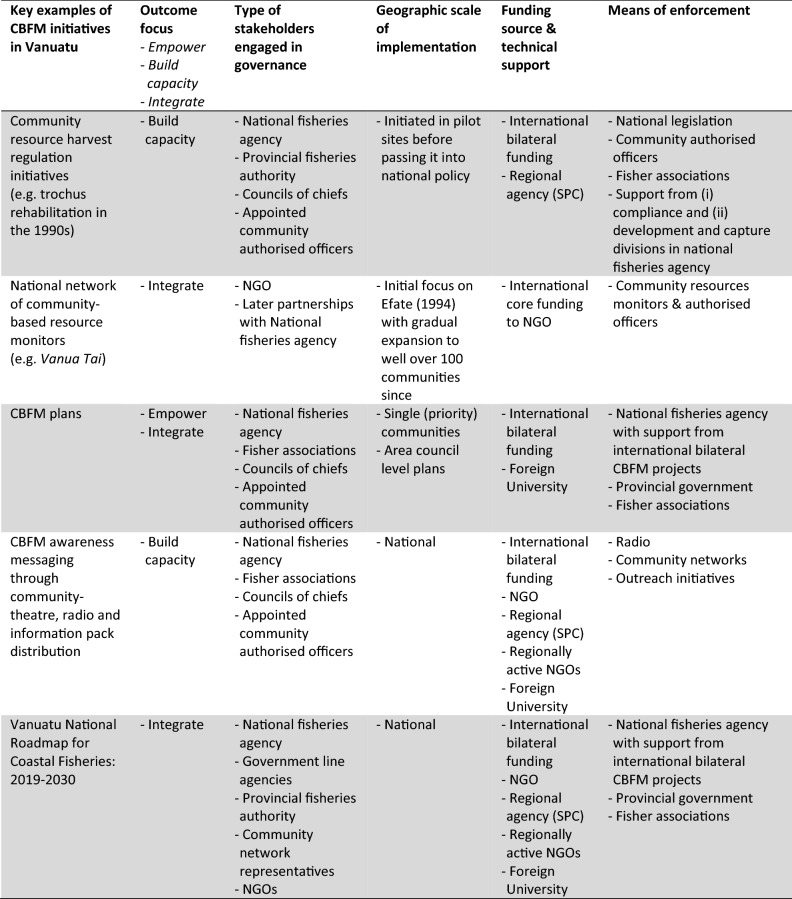
Examples of CBFM initiatives in Vanuatu with their key institutional arrangements. The types of initiatives here provide context to squares depicted in Figs. 2, 4 and 5, including self-initiated community actions (light blue squares) CBFM initiatives emerging from external support to communities (dark blue squares), and interventions that contribute to creating conducive (policy) environments for CBFM (green squares)

## A framework to examine CBFM scaling in Vanuatu

Recent literature emerging around scaling of agriculture innovations reaches a broad consensus that scaling processes are complex, multi-level, non-linear, and ultimately the result of many controlled/intentional and uncontrolled/unintentional inputs into a system (Geels and Raven 2006; Geels 2019). ‘Innovation diffusion’ perspectives in the literature offer useful insights on scaling dynamics (Rogers 2003; Mahajan et al. 2021). These tend to theorise the spread of certain behaviour change and/or technology adoption, with a focus on the subject matter being scaled, rather than examining scaling as a process happening in a broader socio-political and economic environment. Understanding how an innovation interacts with the environment in which it is to be scaled, however, is as important as the inherent ‘quality’ of that innovation. We argue that holistic frameworks offer further insights by incorporating both processes and structural elements as influential to the shaping of the innovation itself and its scaling trajectory leading to a regime shift.

Steenbergen et al. (2022) build on multi-level perspectives (MLP) on socio-technical transitions (Geels 2002) in the scaling agriculture-innovations literature. The theory incorporates multiple processes and structural elements to explain how CBFM can become adopted and practiced at scale. Scaling trajectories are mapped along two main ‘scaling’ axes (Fig. 2): **scales of structuration** as a measure of an innovation’s (horizontal and vertical) integration into a system (i.e. extent by which CBFM is part of ‘how things are done in fisheries management’), and **phases of scaling** as a measure of an innovation’s maturity and its alignment to that broader socio-political environment (i.e. extent by which CBFM is shaped to allow ‘acceptance’, up-take and/or absorption) (Wigboldus et al. 2016).

Using this framing, a society’s resistance to change, or ‘rigidity’, may be described at three scales. The ‘niche’ level represents small spaces that allow introduction and testing of innovations. These may remain peripheral to societal norms and can be rejected with little legacy or risk to the system. At the ‘regime’ level the system is more rigid and so too more difficult for innovations to enter and integrate. Once an innovation is adopted in the regime level, however, it becomes a more permanent part of societal fabric. Lastly, the system is most rigid at the ‘landscape’ level. Norms at this scale represent the fundamentals of the national coastal fisheries context, for example, aspects of governance arrangements (centralised and/or decentralised), physical environment (archipelago or continental coastlines), and/or degree of resource commodification (subsistence utility and/or barter trading and/or market-driven fishing).

Following Geels (2019) we use four phases to illustrate how innovations evolve and are absorbed into a regime. These include **experimentation** as a ‘testing’ phase for new ideas, approaches, and concepts, **stabilisation** of innovations as they go through iterations of experimentation and are refined, **disruption** as innovations gain enough acceptance to influence the broader reconfiguration, and finally, **institutionalisation** as innovations become part of the regime’s reconfiguration. Reflecting on key developments over time, these trajectories are mapped out as contributions by policy initiatives (green squares), by community-oriented interventions involving external CBFM support agencies (dark blue squares) and by community or civil society actions that are independent of external support (light blue squares) (Fig. 2 and see also Table 1 for examples of CBFM interventions).

Several cross-cutting considerations are worth noting when making sense of scaling trajectories. Firstly, an innovation’s evolution is by no means linear, consistent nor uni-directional. External drivers of change that are beyond the scale and scope of any single innovation can significantly affect willingness to invest in refining or applying an innovation, including biophysical influences (e.g. natural disasters, climate change) and human-induced influences (e.g. political unrest, public-health crises). Secondly, regimes, and the systems that constitute them, are path-dependent (Muilerman et al. 2018). Institutionally accepted ways of doing things are habitual and difficult to erase or shift. Thirdly, where scaling initiatives are framed as an inherently positive endeavour, it is important to reflect on potential negative impacts that could arise. Consideration for ‘responsible scaling’ (Wigboldus and Leeuwis 2013) is imperative in ensuring both interventions and planning mitigate negative consequences where these are predictable, or allow for adjustment and adaptation where these appear unexpectedly.

Pivotal to this framework is clarifying explicitly *what is being scaled*, namely CBFM. Unlike a tangible technology that can be picked up, used and passed on, CBFM is an institution; ‘a way of doing things’. It forms around key principles that enable people to act collectively (i.e. represent various interests and aspirations) and to self-adapt management so that local fisheries’ productivity and function is maintained over time and under variable conditions (Armitage et al. 2008). These key principles behind sustainable community-based fisheries include dimensions of knowledge, practice, organisation, and connectivity. People act based on what they know or believe; so **knowledge** may range from notions of being aware that people can affect the health of fisheries, to understanding technical resource management principles. The extent of responsible fishing **practices** (i.e. no destructive fishing, overfishing) indicate people are fishing with a plan and vision of a future, that are resistant to market demand pressures, responsive to emerging threats, and inclusive and fair in the opportunity for access and benefits. **Organisation** refers to how people are organising themselves (i.e. mechanisms for fair decision-making, conflict resolution, and broad representation), for example, with clear understandable rules and regulations in place for which compliance is high and with strong leaders acting with legitimacy. Key here is whether decision-making and access to benefits are both inclusive and subject to appropriate checks and balances on fairness. Lastly, the levels of **connectivity** recognises that local stakeholders (i.e. communities, fishers, and/or fisher groups) operate as part of larger governance structures. Administrative structures may form channels through which people receive support and contribute to national fisheries agendas, whilst socio-cultural governance structures through, for example, customary community networks, may provide social protection. Understanding that people engage their various social, political and/or economic networks to exert influence, coordinate management at ecosystem levels and/or ‘get things done’, indicates the cross-sectoral nature of influence on how innovations are accepted or rejected into daily practices.

This framing requires both direct efforts towards fisheries management on the ground and seemingly indirect efforts that develop ‘enabling’ contexts for CBFM, whilst also ensuring capacity to account for unforeseeable ‘transformative’ events. Scaling interventions therefore operate at a number of institutional and geographic scales, and inevitably are subject to actions that organisations both can and cannot control.

Many community-based initiatives take the establishment of a tangible output, like a local fisheries management plan (representing ‘uptake’), as an end-point for evaluation. In such cases, scaling seeks that objective and can be undertaken without integration with national institutions or investment in national capacity. Although such outputs may be important milestones, equally important is to understand the implementation and use of such outputs, and the social and resource outcomes that flow from them. Continuity beyond reaching material milestones, like management plans in multiple communities, rests on how those outcomes are reflected and built upon within broader regimes.

## Tracing CBFM’S growth in Vanuatu

### Contextualising amongst broader shifts in CBFM-related policies and narratives

**Fig. 3:**
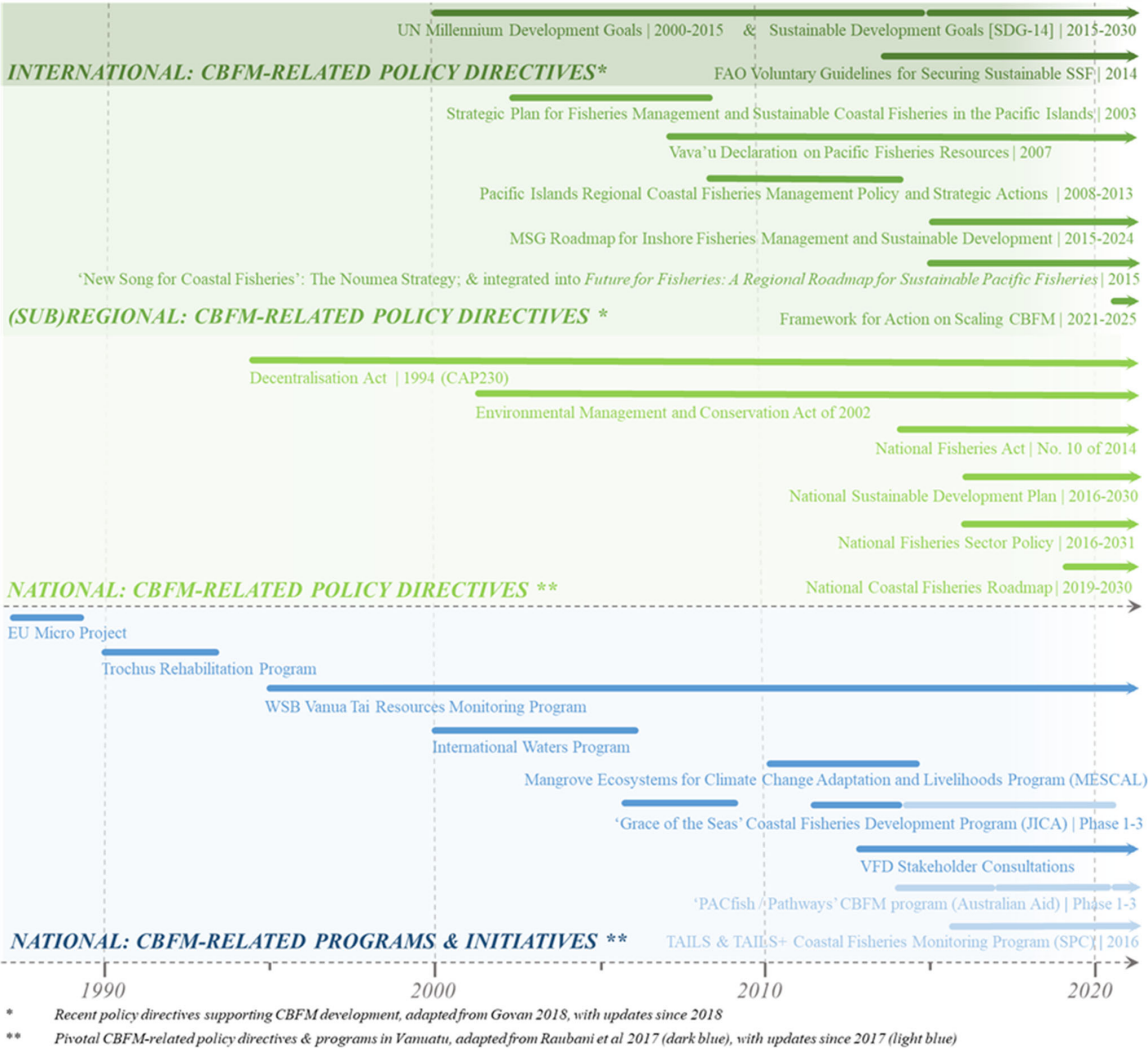
Examples of the pivotal policy directives at international, (sub)regional, and national scale (adapted from Govan 2018) and key projects and initiatives at national scale (adapted from Raubani et al. 2017) described in “Contextualising among broader shifts in CBFM-related policies and narratives” and “Formative CBFM programs and initiatives in Vanuatu” sections, that collectively have contributed to the emergent development of CBFM in Vanuatu

CBFM growth over the last decades in Vanuatu is embedded in policies at multiple levels, from international treaties and agreements to regional, sub-regional and national policy directives (Fig. 3). These policies have coalesced behind the increased momentum of CBFM and are partially the result of grassroots influence and calls for change. Their inclusion here is therefore a recognition of their role as a catalyst for change as much as they are a result of changes happening below them. Below we highlight key policies and agreements that have emerged as a form of endorsement of change, and which have proven pivotal to enabling CBFM agenda-setting in Vanuatu. We examine, in particular, trends towards adoption of decentralisation mandates, socio-ecological sustainability objectives, and a coastal fisheries focus.

Decentralisation trends can in part be mapped at the highest global scale to social justice movements in the 1980s and 1990s (Forsyth 2008), which did much to give indigenous voices a platform of influence. This gained particular traction in the conservation and rural development sectors, wherein transitions away from prescriptive top-down development solutions made way for decentralised, collaborative, and community-based approaches to planning, implementation, and evaluation. This fuelled a reinvigoration of anthropological perspectives on development that put people and place at the centre of the development discourse (Chambers et al. 1989; De Sardan 2005). In parallel, the wave of former colonial territories gaining independence in the second half of the twentieth century meant demand for decentralised modalities for development grew in the lower income countries of tropical regions (Shepherd 1998).

Vanuatu’s independence in 1980 saw the enactment of the national constitution as a sovereign state. Explicit within this was the recognition of *kastom* governance structures through the establishment of a national council of chiefs with ‘a general competence to discuss all matters relating to custom and tradition’ (Republic of Vanuatu 1980, p. 12). The 1994 Decentralisation Act formalised levels of government and established the foundation for decentralised government support, from national down to area level. Besides government rule taking shape in these early decades of independence, civil society groups too became more active and organised. The emergence of the *Vanua-Tai* network in 1995, for example, started as a relatively small community-based network of turtle monitors around Efate Island (Hickey and Johannes 2002). It has since developed into a national network of natural resource monitors with continuous funding support and increasing collaboration with government agencies like the Department of Environment Protection and Conservation and VFD. Such networks have served as important conduits for civil society action and mobilisation of communities across dispersed and remote islands.

Catalysed by a global realisation of human impact on the environment, finiteness of resources, and of course climate change, the interface between societies and the environment has become core to development discourse (Allison and Ellis 2001; Ellis and Biggs 2001). Vanuatu’s ‘Peoples Plan’ endorsed in 2015 provides the nation’s benchmark targets and strategic direction for sustainable development, across all ministerial programmes.

In the Pacific, fisheries have always formed a central cornerstone of the region’s development agenda. Whilst much of the regional focus has traditionally been on tuna, coastal fisheries gained more standing around the same time the Voluntary Guidelines for Securing Sustainable Small-Scale Fisheries (FAO 2015) appeared. In the Pacific, significant regional documents included the Vava’u Declaration on Pacific Fisheries Resources (Pacific Islands Forum 2007), the Pacific Islands Regional Coastal Fisheries Management Policy and Strategic Actions 2008–2013 (the ‘Apia Policy’), and two more recent initiatives, the ‘New Song for Coastal Fisheries’ facilitated through the Pacific Community (which was integrated into the ‘Future for Fisheries: A Regional Roadmap for Sustainable Pacific Fisheries’) and the ‘Roadmap for Inshore Fisheries Management and Sustainable Development 2015–2024’, facilitated through the Melanesian Spearhead Group (Melanesian Spearhead Group 2015) (see Govan 2018 for reviews of this evolution; Karcher et al. 2020). In 2021 the endorsement of the ‘Pacific Framework for Action on Scaling up CBFM’ (Pacific Community 2021, p. 2) operationalised these broader policy directions by “provid[ing] guidance to PICTs in developing, prioritising, and implementing national actions” that will support domestic ambitions to spread co-management capacity across their coastal fisheries constituencies.

Coastal fisheries policy in Vanuatu materialised relatively late, following a strong environmental conservation movement up until the 2000s that extended to coastal marine spaces. Prior to the enactment in 2014 of the current, revised National Fisheries Act (this follows from earlier revisions including Cap315 of 2005 and Cap158 of 1982), governance over coastal resources and areas was less defined and practically fell to environmental management (under the 2002 Environmental Management and Conservation Act), since fisheries management at the time was strongly ocean-focussed. Whilst the Act makes mention of coastal fisheries resources, the Fisheries Sector Policy endorsed 2 years later offers specific attention to the dependence on coastal fisheries by ni-Vanuatu, and by Vanuatu’s domestic economy as a whole (MALFFB 2016). This, furthermore, led to the endorsement in 2019 of the National Coastal Fisheries Roadmap, which sets out a 10-year strategic direction for development of coastal fisheries (Vanuatu Fisheries Department 2019). The Roadmap provides the most up-to-date policy mandate for CBFM in Vanuatu, resulting from the increasing regional and domestic momentum behind CBFM.

### Formative CBFM programmes and initiatives in Vanuatu

In their summary of CBFM-related initiatives prior to the mid-2010s, Raubani et al. (2017) identify seven influential initiatives that shaped coastal fisheries development in Vanuatu. In doing so, they elucidate the main drivers behind a rise in co-management, primarily through external bilateral funding. These trends show progression from early, predominantly direct interventions in fisheries towards multi-sectoral approaches that incorporated different stakeholders and targeted different levels of governance to achieve both local, immediate impact and structural, longer-term change.

Starting in the late 1980s, the first large scale community-based initiatives engaged through production-bolstering interventions which sought to develop local entrepreneurial capacity. The ‘EU micro project’, for example, provided equipment and technical skills and was the first concerted investment into community level fisheries (Raubani et al. 2017). Subsequent initiatives in the early 1990s recognised the need for simultaneous policy interventions. The first to make meaningful steps was a series of projects aimed towards trochus (*Trochus niloticus*) rehabilitation in coastal areas, which highlighted the importance not only of legislative tools to control harvest and legitimise management measures, but also the engagement of community stakeholders to enforce those (Amos 1993; Jimmy 1995). Subsequent programmes run by VFD drew from this approach, with further investments into, for example, developing national management plans for various species (2010 onwards), refinement of coastal fisheries regulations (2014 and 2016), and establishment of strategic planning tools like the coastal fisheries roadmap (2019).

By the end of the 1990s the *Vanua Tai* community-based resource monitoring network was highly regarded amongst domestic stakeholders for its increasingly extensive reach within communities. By 2003, 100 coastal communities were represented in the network that included over 200 resource monitors (Johannes and Hickey 2004), and were forming partnerships with various government departments to implement programmes. In the process, many monitors voluntarily assumed additional roles, including, for example, as authorised fisheries officers. In doing so, these individuals embodied VFD’s furthest governance reach into communities. As noted by Hickey (Vierros et al. 2010, p. 33) in his reflection on linkages between local, traditional, and national government resource management in Vanuatu, “once the underlying rationale of fisheries regulations […] are understood […], community leaders are happy to include these in their village-based management regimes. This has boosted the importance and value of cooperative management principles”.

Not only was there an expansion in stakeholder engagement to lower-level civil society networks, but also upwards at a regional level. Multi-country funding initiatives for coastal fisheries saw significant investment distributed amongst the Melanesian countries and other Pacific countries. The International Waters Program (2000–2006; involving 14 PICTs), and the Mangrove Ecosystems for Climate Change Adaptation and Livelihoods (MESCAL; 2010–2015) were important regional investments, with national and local nodes in each country. Amongst other changes, these brought about increased marine conservation area designation, community capacity building, ecosystem management approaches, and climate change mitigation programmes in Vanuatu (Raubani et al. 2017). It also engaged Vanuatu in increasingly active regional-level discussions on coastal fisheries development after 2010, mostly through facilitation by what is now known as the Fisheries, Aquaculture, and Marine Ecosystems division at the Pacific Community (SPC-FAME). A regional coastal fisheries production monitoring programme, ‘TAILS’ (Pacific Community 2019) was, for example, piloted with SPC-FAME in 2016. It led to the rollout of data collection on tuna and reef fish catch from small-scale fishers in remote locations (Pacific Community 2019), of which later refinements produced a tailored Vanuatu version, ‘TAILS+’.

Around 2010 Vanuatu also saw the start of its first long-term, continuous investments into coastal fisheries development. Whilst prior bilateral project funding for CBFM at VFD never exceeded about 10 million Vatu per year (~ 87 500 USD), the following decade saw this increase to an estimated average annual investment through bilateral projects of over 40 million Vatu (~ 350 500 USD). Three main investments have since been through several phases and are still operative today. The first being the Grace of Seas Program, supported through the Japan International Cooperation Agency, starting in 2008 and running until the present. The second being a string of smaller research for development projects under the French National Research Institute for Sustainable Development, which provided technical management support to VFD around participatory fisheries area management. Finally, investments by the Australian Government, starting 2014 and continuing to the present.

As part of their shared mandate to strengthen co-management of coastal fisheries in Vanuatu, these initiatives helped establish collaborative partnerships between VFD and key coastal communities (Nimoho et al. 2016; Gereva et al. 2021). Under these participatory management programmes, CBFM’s orientation at VFD also shifted from the previous environmental-focus to a more rural development-focus, where issues of income and livelihoods, food-security and empowerment gained equal importance as outcomes next to ecosystem performance indicators. At the same time, these programmes were increasingly shaped from one phase to the next by strengthened VFD. These bilateral partnerships further refined VFD’s co-management approaches, to the point that CBFM has come to form the prime national implementation strategy for supporting communities and their coastal fisheries.

### Examining CBFM growth through innovation pathways

**Fig. 4:**
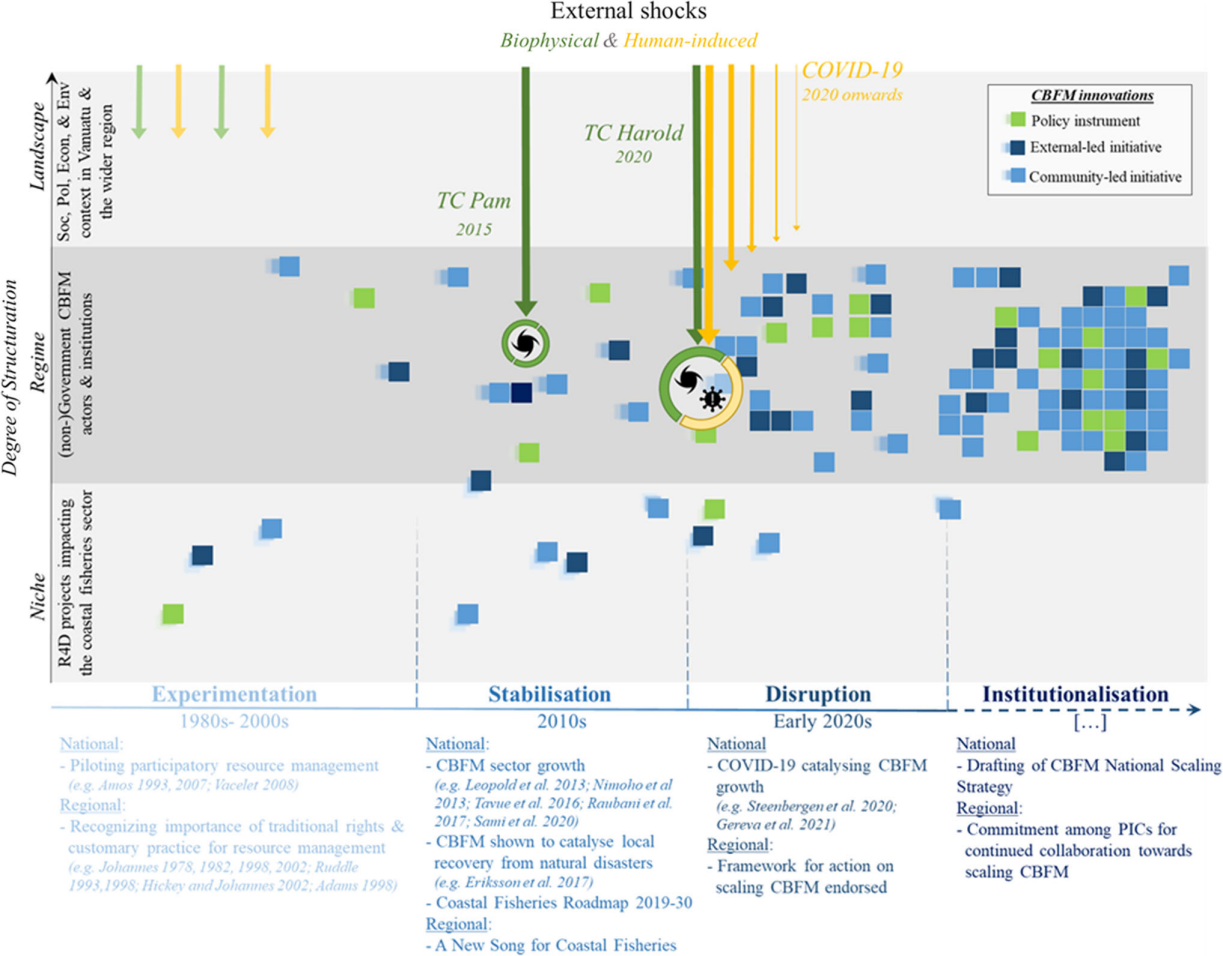
CBFM scaling framework, adapted from Steenbergen et al. (2022), using the dimensions of ‘scales of structuration’ and ‘phases of scaling’ to sense-make the different innovation trajectories of CBFM that have contributed to a regime reconfiguration supporting institutionalisation of CBFM (we draw here from selected key literature related to CBFM in Vanuatu, including: Malinowski 1922; Johannes 1978, 1982, 1998, 2002; Amos 1993; Ruddle 1993, 1998; Hickey and Johannes 2002; Vacelet 2008; Adams 2012; Léopold et al. 2013; Tavue et al. 2016; Eriksson et al. 2017; Raubani et al. 2017; Andrew et al. 2020; Sami et al. 2020; Gereva et al. 2021; Steenbergen et al. 2022)

Here we examine the coastal fisheries regime reconfiguration thus far in Vanuatu, by tracing the CBFM innovations trajectories across scaling phases. Our analysis indicates how CBFM as an innovation has developed through phases of experimentation, stabilisation, and disruption, so that Vanuatu finds itself at a critical time of transition towards institutionalisation of CBFM (Fig. 4).

Prior to the **experimentation** phase of CBFM, anthropological works of the early twentieth century (e.g. Malinowski 1922) laid the foundation for wider recognition of traditional management structures in Melanesia. Later works by, for example, Ruddle (1993, 1998), Johannes (1998, 2002) and Hickey (2008) drew such insights into resource and fisheries management, and into the Vanuatu context of *kastom*. These academic perspectives materialised into practical forms of interventions in small pilot projects like the government-driven initiatives behind trochus rehabilitation (Amos 1993), the emergence of the *Vanua-Tai* resource monitoring network (Hickey and Johannes 2002) and later in the form of foreign aid funded research for development initiatives. As reported by Dumas et al. (2012, p. 1) in their national review of coastal area management effectiveness, Vanuatu experienced “since the early 1990s a striking upsurge in small-scale initiatives to protect marine invertebrate resources […]. One of the most common actions was the creation of very small, village-based marine reserves (tabu areas) with permanent or temporary fishing bans for invertebrate species”. They make further reference to ‘booming’ of smaller community-based management trials in Vanuatu in this period. These smaller experimental forays into co-management served as an important, somewhat crude, refining period where key principles of collaborative management, partnership building and cultural awareness, were first married with technical insights of fisheries management (Vacelet 2008).

By 2010 approaches to fisheries co-management were becoming more sophisticated and increasingly received mention in national policy and development discourse in Vanuatu (Léopold et al. 2013). The previously mentioned set of long-term investments into Vanuatu coastal fisheries by bilateral projects, formed the second generation of innovation-building that drove CBFM into a **stabilising** phase. Indicative of CBFM’s expanded applications was its integration as part of disaster relief efforts. When Tropical Cyclone Pam struck in 2015 many coastal communities were affected. Human capital, collaborative networks, and community-based organisational capacity developed as part of CBFM projects proved valuable in increasing communities’ ability to deal with those disruptions independently (see Eriksson et al. 2017 and Pakoa et al. 2019 for examples of CBFM-related coping strategies that communities applied).

The stabilising phase of the 2010s was furthermore supported by a period of close collaboration with regional institutions like SPC-FAME. The former VFD director of 12 years was director of SPC-FAME from 2014 to 2019, and a cohort of ni-Vanuatu fisheries officers worked there at the time. The ‘New Song for Coastal Fisheries in the Pacific’ found particular traction in Vanuatu, resulting in aligned domestic developments like the formulation of the National Roadmap for Coastal Fisheries in 2019.

The phase of **disruption** that saw CBFM further cement its place in Vanuatu’s coastal fisheries sector, culminated with the acute international shifts and changes brought about by the COVID-19 pandemic. With strict border closures from March 2020, Vanuatu was effectively closed off with no outbreak of note in the first 2 years. Foreign technical aid continued only through programmes with established in-country teams and structures. At all governance levels Vanuatu institutions and their actors shifted towards increased self-coordination (HAG and VANGO 2020). At community levels this saw the resurgence of traditional (*kastom*) practices, to make ends meet following the collapse of local economies dependent on external industries, like international tourism (Movono et al. 2021). At a national level coastal fisheries management relied on management capacity and collaborative partnerships developed under various CBFM initiatives (Gereva et al. 2021).

At no point was this ‘new reality’ made clearer than at the beginning of the pandemic when the Vanuatu government was forced to deal with the impacts of a second category-five tropical cyclone in 5 years, with Tropical Cyclone Harold in April 2020. The response was in stark contrast to five years prior during Tropical Cyclone Pam, when there was a massive inflow of external aid support, often misplaced or misdirected. During Tropical Cyclone Harold, CBFM proved a critical asset to communities as many were forced to address the immediate aftermath of the cyclone independently, following delays in relief support reaching people under COVID restrictions (Steenbergen et al. 2020). From 2020 onwards VFD’s response to community fisheries support under COVID-19 saw a strong pivot towards CBFM approaches as a conduit channel for support to the ground.

In this same period the Regional Scaling Framework was endorsed (2021). This framework emerged from extensive deliberation and a series of consultative stages, through regional and subregional fora involving stakeholders from the 22 PICTs, but also from technical fisheries and development institutes (Lalavanua et al. 2021). The resulting document set out proposed building blocks for member countries to develop their own national scaling strategies. In Vanuatu, this energised and informed a series of drafting workshops at VFD in 2021 and 2022 for the development of a National CBFM Scaling Strategy. Next to the National Coastal Fisheries Roadmap of 2019, this CBFM scaling policy directive can further clarify CBFM as VFD’s primary delivery pathway for coastal fisheries support. In so doing, it edged CBFM towards **institutionalisation** in Vanuatu. In the following section we examine what this may look like.

## Discussion: Institutionalising CBFM through national coordination

The strategic approaches and actions highlighted in the Regional Scaling Framework (Lalavanua et al. 2021) identify the critical elements that PICTs and regional experts consider necessary to create national programmes for CBFM. It is explicit in not providing a blueprint solution, understanding that national programmes vary depending on the extent of their engagement with CBFM and their particular cultural and governance context. A national CBFM programme can be seen as the pragmatic materialisation of CBFM scaling thinking and a result of translating key CBFM principles into action. Here we frame it to be a sum of many initiatives seeking to develop and support diverse and dispersed constituencies of coastal communities to practice CBFM. Given the previously outlined growth of CBFM in Vanuatu towards regime disruption, the establishment of a national CBFM programme implemented across coastal fisheries may consolidate government commitment and advance CBFM innovation-scaling to institutionalisation in Vanuatu’s coastal fisheries.

As has been illustrated, CBFM institutions need to balance various competing interests, so although similar approaches may be used to support, strengthen, or create these institutions, very different institutional arrangements may be needed for meaningful, sustained change at scale. In some places CBFM institutions already exist that help people govern and manage fisheries resources (e.g. within customary systems of fishing closures, like Tabu areas). Whilst such cases may need nurturing or orienting to a contemporary fishery sustainability purpose, in other places management systems need to be reinvigorated or developed (e.g. communities where fishing is unregulated and driven by strong market demand). A national programme may therefore take form in Vanuatu depending on, for example, the nature of coastal tenure, the capacity and capabilities of national and provincial agencies, strength of policies, and supportive civil society organisations.

**Fig. 5:**
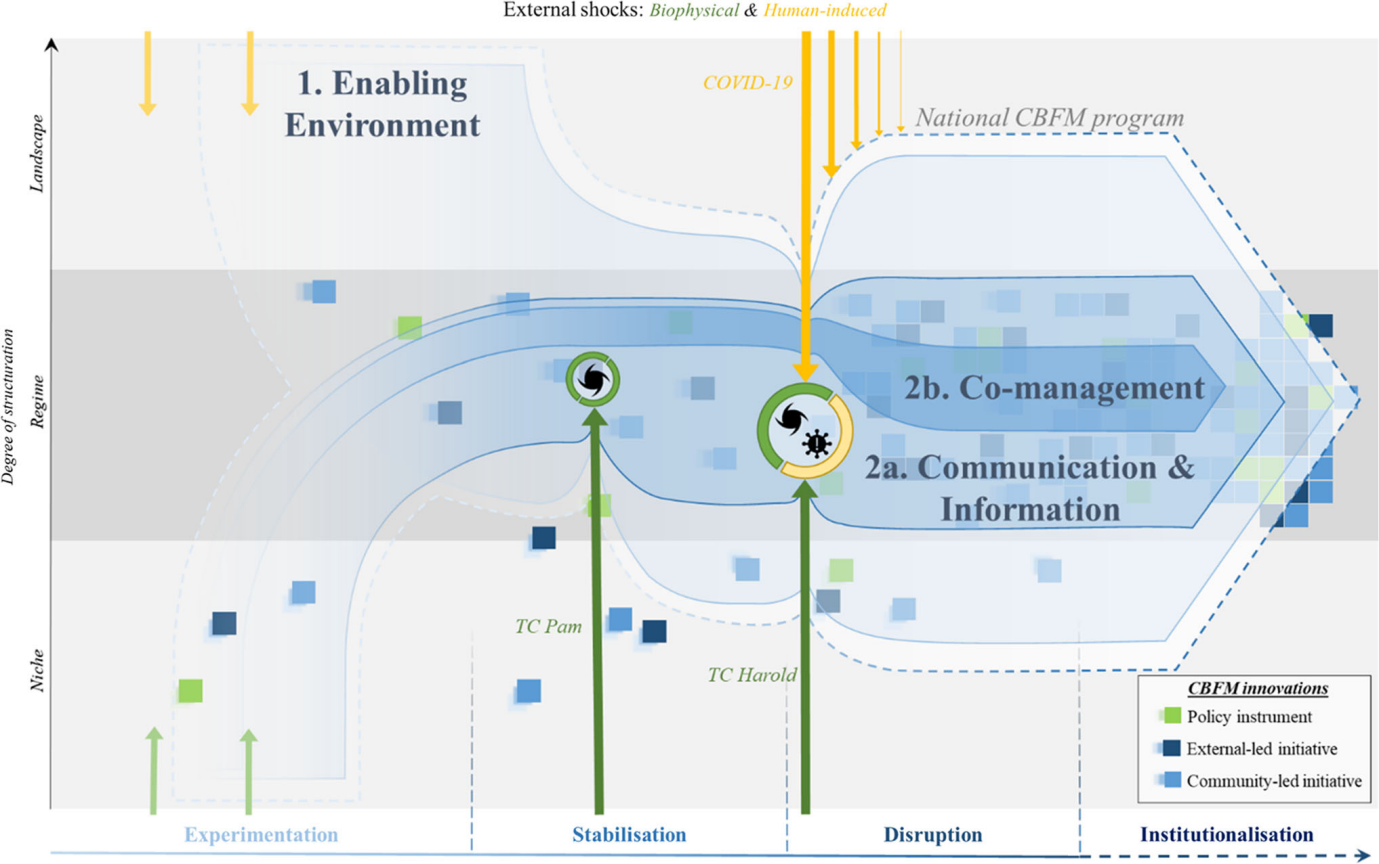
CBFM scaling framework depicting the three action pathways that consolidate past and current efforts to institutionalise CBFM in Vanuatu

So, in drawing from Vanuatu’s institutional context and from guidance in the Regional Scaling Framework, in addition to the long experiences of CBFM supporting institutions, like the Locally Managed Marine Area network (Govan et al. 2008, 2011, 2015), we broadly categorise the necessary strategic direction and coordination of investments and interventions in coastal fisheries. We identify three intervention pathways that could collectively form a national programme (Fig. 5): Pathway-1 involving actions that foster an enabling environment for uptake of CBFM (e.g. CBFM supporting policy and regulations); Pathway-2a involving direct actions that are broad reaching across large constituencies (e.g. information broadcasting and awareness campaigns); and Pathway-2b involving direct actions that are more focussed, resource-intensive, and targeted towards smaller targeted constituencies (e.g. site based co-management interventions). Whilst many activities under these categories are already happening, and have contributed to the regime reconfiguration thus far, a national programme functions to consolidate and coordinate (i.e. institutionalise) them under an overarching logic and theory of change.

Pathway-1, ‘enabling environment’, focuses on indirect supportive measures that contribute to creating broader conducive conditions under which CBFM can spread and strengthen. Ayers and Kittinger (2014, p. 260) usefully reflect on conditions that promote co-management, and refer to the importance of a “creation story of governance arrangements”, something this paper presents. They further outline key areas of inquiry that require integration in policy and planning practice, including the framing of what a ‘community’ is, understanding legitimacy of process, and recognising time frames and costs associated with regime shifts. Whilst smaller CBFM investments have often sought immediate impact in communities, and therefore invested in ground-level, highly visible activities, enabling measures seek more structural, long-term change. As such, they are often less immediate and visible during implementation. Measures under this pathway may, for example, include developing and enacting national policies and legislations, implementing provincial ordinances and strategies, and strengthening institutions’ capacity, and building political momentum. VFD as the national fisheries agency, forms a central target entity, with its sub-national line agencies and authorised bodies. Non-government actors, including civil society groups, non-government organisations, and universities, are equally important in amplifying the government coordination and support to constituencies that are beyond the reach of government departments for whatever reason.

Pathway-2a, ‘communication and information’, is one of two pathways of direct CBFM action, whereby activities here focus on extensive reach. This may include both broadcasting information and creating fora for information exchange. Information broadcasting would seek to ensure a maximum range of people have access to information on, for example, ecology and fish biology to aid an understanding around connections between human activity and state of stocks, or be well informed of how to access information or support (e.g. be aware of national rules and regulations and know who to contact for support), or better still mobilise local capacity to act (e.g. have capacity to identify threats or problems and decide what action to take to address it). Priority of information exchange for Pathway-2a would be to promote dialogue amongst stakeholders across scale for feedback, contextualising, and discussion (Crona and Bodin 2006; Armitage et al. 2008). This could better inform policy, identify barriers to progress, and strengthen context-appropriate management understanding and capacity. Such action may focus on drawing in the most active or productive coastal fishing communities, and concerned, interested or active fishing stakeholders.

Pathway-2b, ‘co-management’, involves the most direct on the ground action with communities, and given it is resource intense and costly, would have the least extensive coverage. Activities and interventions here would aim to establish collaborations that achieve action, co-learning and rules towards sustainable coastal resource management and wellbeing. These actions effectively form pockets for niche innovation and experimentation to allow continuous adaptation to CBFM (Shilomboleni and De Plaen 2019), so it remains relevant and effective. With its most narrow reach, such activities are likely to target select coastal fishing communities that are most motivated or most in need of support.

### Tracking performance of a national CBFM scaling programme

The effective implementation of CBFM will require systematic and ongoing monitoring of progress towards achieving the national vision for coastal fisheries as stipulated in Vanuatu’s coastal fisheries roadmap. Critically, this needs to occur across investments from both national budgets and bilateral partnerships, and be integral to implementation processes, rather than an afterthought. It, furthermore, requires a threefold approach involving coordinating of various investments to ensure their complementarity as inputs into the system along the three main action pathways, live monitoring of implementation and actions, and capturing outcomes of those actions and how they feedback to inform the national programme’s coordination.

Mixed methods approaches that employ both quantitative and qualitative metrics and methods are required to capture the complexity and diverse social and ecological objectives of a national programme of CBFM (see also Visser 2004; Boyd and Charles 2006). For this, existing initiatives can be utilised to measure outcomes of scaling efforts by, for example, consolidating the various data collection programmes that VFD already has in place, like monitoring initiatives on catch-effort in community fisheries, domestic fish trade and distribution, and licensing. Results from these can quantify the extent of active CBFM on the ground, whilst qualitative data through reporting mechanisms by national and provincial staff can usefully qualify such results.

## Conclusion

Vanuatu has a long history of engagement and innovation in community approaches to coastal fisheries management. This body of research and practice has, until recently been implemented as a series of projects and investments, mostly by bilateral partners in partnership with national agencies. During the last decade, this stream of investments has coalesced into a programme of field and policy-based work led by VFD. As progress has accelerated VFD has invested in new structures and processes to underpin the need to act in more places and at larger scales (see also Pigford et al. 2018). Critically, also, VFD has provided a vision for how CBFM can contribute to national food security and rural development goals.

Steenbergen et al.’s (2022) theory of scaling for CBFM has provided a lens to better understand the evolution of CBFM in Vanuatu, from small niche research-for-development projects that shaped CBFM during the experimentation phase (see also Shilomboleni and De Plaen 2019) to mainstream application. Specifically, it identified disruption points in this history and aided recognition of three primary pathways for influencing change. We used the theory of scaling for CBFM to clarify the pathways, and to highlight the interplay of structures and processes that shape the innovation on one hand and the environment or regime that CBFM functions within on the other. This translates, in practical terms, to the need to move away from mosaicked landscapes of siloed projects and actor-groups. Instead, coordinated programmatic approaches that map how different actors and their actions can address priority areas, are required for effective institutionalisation of CBFM. The emergence of a national programme has meant that priorities for investment and ways of doing things have changed fundamentally as the challenges and institutional landscape have evolved.
